# Animal empathy reconsidered: a multidimensional profile account

**DOI:** 10.1002/brv.70196

**Published:** 2026-06-28

**Authors:** Albert Newen, Maja Griem, Ludwig Huber, Thomas Bugnyar, Aaron Blaisdell, Simone Pika

**Affiliations:** ^1^ Institut für Philosophie II Ruhr‐Universität Bochum Universitätsstr. 150 Bochum 44801 Germany; ^2^ Messerli Forschungsinstitut Veterinärmedizische Universität Wien Veterinärplatz 1 Vienna 1210 Austria; ^3^ Department für Verhaltens‐ und Kognitionsbiologie Universität Wien Althanstraße 14 Vienna 1090 Austria; ^4^ Department of Psychology University of California Los Angeles 1285 Franz Hall, Box 951563 Los Angeles CA 90095 USA; ^5^ Institut für Kognitionswissenschaft Universität Osnabrück Artilleriestrasse 34 Osnabrück 49076 Germany

**Keywords:** animal cognition, animal empathy, comparative psychology, empathy, multidimensional profiles, profile account, species‐sensitivity, varieties of empathy

## Abstract

Empathy is the glue that holds societies together and yet several fundamental questions about empathy persist. What is empathy (the definitional question)? Is it uniquely human and, if not, which nonhuman animals possess empathy (the distribution question)? Which type or quality of empathy is realized in different species (the quality question)? To tackle these three questions, we developed a species‐sensitive, multidimensional profile account of empathy. The main function of this account is to enable cross‐species comparisons and to capture the rich variety of typical empathetic phenomena. Therefore, we aim to characterize behaviour‐based cognitive profiles of empathy which are built on multifactorial characterizations of the dimensions of empathy and of the features realizing these dimensions. The distribution question can be answered by assessing family resemblances of profiles of empathy to paradigmatic cases of empathy. Answers to the quality question can be provided with reference to the relevant empathy profile, which allows us to describe and predict associated behaviours. To gain an initial understanding of the feasibility of this framework for interspecies comparisons, we applied it to four groups of animals: rodents, apes, canids and corvids. Comparing these groups, we demonstrate that each species has a specific empathy profile which has a predictive power: in complex situations requiring empathy, distinct profiles will result in more distinct behavioural responses whereas similar profiles will result in more similar responses, even among phylogenetically distinct groups. This new multidimensional profile account enables fine‐grained comparisons within and between species instead of the prevailing all or nothing perspectives of empathy. Furthermore, it offers the integration of phylogenetic and ontogenetic perspectives thereby providing a crucial tool to explicate the notion of empathy to humans and other animals in a species‐sensitive way. We demonstrate this framework by applying it specifically to empathy, and the framework's advantages invite it to be generalized to all rich and flexible cognitive abilities in nonhuman animals.

## I. INTRODUCTION

There is still no consensus as to which nonhuman animals share empathy with us, but there is increasing evidence that “empathy has the backing of a long evolutionary history” (de Waal, 2009, preface). Indicators include many commonly accepted cases of prosocial behaviour directed towards other's affective states, e.g. in chimpanzees (*Pan troglodytes*) (Boesch *et al*., 2010; Greenberg *et al*., 2010). But we also observe highly debated behaviours, e.g. the cage‐freeing behaviour of companion rats (*Rattus rattus*; *Rattus norvegicus*) which we will discuss in Section V.6.a (Bartal, Decety, & Mason, 2011). Such cases are challenges for any account of empathy.

What exactly is empathy and do nonhuman animals have it in their cognitive and behavioural repertoire? This question is at the heart of *the definitional challenge*: the existing body of work does not provide a commonly agreed definition of empathy. A pessimistic evaluation is that “there are probably nearly as many definitions of empathy as people working on the topic.” (de Vignemont & Singer, 2006, p. 435). However, in a recent meta‐review Eklund & Meranius (2021) argued that scholars are moving towards a consensus and characterized four themes in most empathy conceptualizations: understanding, feeling, sharing, and self–other differentiation. Their proposed definition states that “empathy is to understand, feel, and share what someone else feels, with self–other differentiation” (Eklund & Meranius, 2021, p. 306).

However, this definition is too narrow: *sharing what someone else feels* is not involved when Anne empathizes with Peter who is angry because, in his eyes, he underwent a radically unfair treatment; when Anne thinks that the treatment is not really unfair, she may not be angry, but nevertheless feel sorry for Peter who is angry due to an over‐interpretation. Also *feeling what someone else feels* can be lacking in cases of cognitive empathy: a professional anaesthetist can *register* that a patient is in fear to receive the injection by perceiving their fearful face but need not *feel* their fear to do the job by allaying their fear and starting the medical treatment. Furthermore, the notion of ‘understanding what someone else feels’ is radically underdetermined. Finally, we want to go beyond these aspects of registering the other's situation by also investigating an affective attitude of the observer. This is accomplished by also including aspects of response behaviour directed towards the observed person. Thereby, we propose a wide notion of empathy which includes sympathy and compassion as well as empathy‐based response behaviour. The differences between types of other‐directed affective phenomena can be modelled within out framework, but this is not the focus of this article.

Thus, although we accept the meta‐observation of the relevance of these four themes (understanding, feeling, sharing, and self–other differentiation), we propose to use them only as a loose criterion of adequacy for a systematic characterization of empathy instead of aiming for yet another definition involving necessary and together sufficient conditions for empathy. More promising is to aim for a multidimensional characterization of the phenomenon of empathy in such a way that we can analyse the variety of phenomena, with central dimensions leading to adequate descriptions of relevant phenomena and constraints for underlying mechanisms. Thus we no longer aim for a classical definition of empathy, but we start with a minimal working definition and then we propose an account with five key dimensions. These involve registering (*i*) the other's affective state, (*ii*) the other's situation and (*iii*) the other's cognitive states (aka. cognitive perspective‐taking). And, concerning the behavioural response, we propose the dimensions of (*iv*) flexibility and (*v*) social coordination. Each dimension is characterized by a sequence of features that typically implement a given dimension. We selected these dimensions based on categories that frequently appear in literature as being important and closely related to the concept of empathy. Of all the aspects of empathy, understanding the feelings of others is probably the least debated (Eklund & Meranius, 2021). Building on this, we developed the three dimensions of registration. In order to fully understand how someone else feels, it is necessary to register the other's affective state, the other's situation, and the relevant cognitive states. Furthermore, we introduce the dimension of behavioural flexibility which is based on the idea that basic empathetic behaviour, such as emotional contagion, needs to involve some flexibility in order to be adaptive (Nakahashi & Ohtsuki, 2015). Furthermore, we propose to include social coordination as a dimension since we use a wide understanding of empathy, including the response behaviour, and prosocial response is commonly thought to be a strong evolutionary factor driving empathy (de Vignemont & Singer, 2006; Kang *et al*., 2025). We argue that this is an adequate framework for a species‐specific comparison, but we are aware that this general framework would need more fine‐grained distinctions to account for aspects of human empathy (Maibom, 2019) and outline later how those can be integrated into our framework. This is also our strategy to deal with the *methodological challenge*:

How can we assess which other species are able to realize empathetic behaviour (the distribution question) and which type or quality of empathy is realized in different species (the quality question)? Why can we not start with neural correlates of empathy and rely on an inference of neural analogy, that is, the same neural processes imply the same mental processes? Despite impressive progress, a sufficiently clear neural correlate of human empathy has not yet been discovered. First, there are different candidates: a standard distinction is that of affective empathy (involving affective reactions to the other's affective state) and cognitive empathy (to take the mental perspective of others concerning their affective situation) (Shamay‐Tsoory, Aharon‐Peretz, & Perry, 2009). Affective empathy recruits the insula, the brainstem, the inferior parietal lobule, thalamus (Nummenmaa *et al*., 2008) and medial orbitofrontal cortex (OFC) (Hynes, Baird, & Grafton, 2006), while in contrast cognitive empathy relies more on the inferior frontal gyrus, the supramarginal gyrus and the superior temporal sulcus (Hooker *et al*., 2010) (for an overview, see Cox *et al*., 2012). Some scholars suggested a threefold distinction of forms of empathy (Keysers & Gazzola, 2018): strongly bottom‐up driven cases involving emotional contagion; intermediate level types of empathy, such as registering the other's pain and initiating a helping behaviour; and high‐level, more top‐down driven cases, like the sympathy shown when registering the other's anger without oneself being angry but nevertheless being motivated to support the person to assuage their anger. The neural correlates in these three cases differ: in emotional contagion, mirror neuron related processes are intensely involved (Keysers & Gazzola, 2018) while intermediate level cases of empathy seem to involve mainly the anterior insula and rostral cingulate cortex (rCC) (Singer *et al*., 2004; Wicker *et al*., 2003). Most complex top‐down driven cases of empathy seem to especially rely on the neural network of perspective‐taking including especially the temporal parietal junction (Lamm, Bukowski, & Silani, 2016). In sum, there is currently no unitary neural correlate of empathy.

Second, even if we admit the lack of a unitary neural empathy network, we may propose one for each type of empathy (e.g. affective *versus* cognitive empathy). But this cannot be observed in the reported two different ways of classifying human empathy. Third, even if we reached a consensus about the neural correlates for human empathy, then, up to now, the networks remain rather unspecific and the relevant brain areas involved in the network remain rather coarse‐grained and underdetermined due to their multiple functional roles. Furthermore, and most importantly, even if a clear neural correlate with a specific functional role was identified, we would still be faced with the open challenge of transferring this to cases of species with very different brain organizations from those of humans such as birds (Jarvis *et al*., 2005) and insects (Winding *et al*., 2023). Thus, to investigate empathy comparatively across all known species and taxa, using an inference based on neural analogy is not an adequate strategy.

As a new strategy, we develop a ‘theory‐light’ approach (Birch, Schnell, & Clayton, 2020) which consists of several steps, starting with a standard example of human empathy. Based on this, we describe the central dimensions involved, thereby enabling the characterization of typical behavioural and cognitive profiles connected with all varieties of human empathy. Subsequently, we aim to substitute (if necessary) the central dimensions of the human case with dimensions which are applicable to other animal species, i.e. they should be empirically testable without linguistic communication and further developed to avoid anthropocentrism. The result is the multidimensional profile account of empathy that enables us to characterize behavioural and cognitive profiles with regards to empathetic behaviour for any given species. This account involves pinpointing a range of typical dimensions and features implementing these dimensions (prototypical implementations) to systematically describe ideal cases of empathy. Furthermore, it enables us, based on family resemblance relations, to situate less typical cases and to distinguish different affective phenomena. This is inspired by similar methodologies developed in a characterization of emotional episodes (Newen, Welpinghus, & Juckel, 2015) and in a description of profiles of animal consciousness (Birch *et al*., 2020; Dung & Newen, 2023). Our account involves a systematic interdisciplinary perspective to combine relevant insights and perspectives from developmental psychology, primatology and animal cognition as well as philosophy of mind. We hope that this theory‐light approach of describing empathy profiles based on behavioural and cognitive abilities will nurture and be complemented by theory‐heavy approaches which aim to describe the underlying neural implementations. This goes together with the aim to ideally disclose the similarities of the different neural processes underlying similar behavioural and cognitive profiles for different species and thus ideally discover unitary computational processes across species with different brain organizations. Whether there actually are such unitary processes, however, remains an open question. Whether the proposed multidimensional profile account may prove useful and sustaining for fostering a comparative perspective will also be answered by the progress of science (Dung & Newen, 2025).

In Section II, we will set the stage for the new account by using a standard example from our human life to characterize three core aspects of full‐blown empathy. To enable comparisons to other animal species, we then pinpoint five dimensions (as substitutes of the three core human aspects) which can be empirically measured in nonhuman animals living in captive settings or their natural environments. In Section III, we argue that the two currently predominating proposals of animal empathy, the Russian doll model (de Waal & Preston, 2017) and the combination model (Yamamoto, 2017), need to be improved, or at least expanded. To do so, we introduce in Section IV the multidimensional profile account consisting of five dimensions with each dimension being implemented by several typical features of varying complexity. In Section V, we then apply this model systematically to results of recent studies on different nonhuman animal species to demonstrate its explanatory fruitfulness. In Section VI we explicate the status of the new account by describing its characteristics and advantages.

## II. TWO CENTRAL EXAMPLES AND A NEW THEORETICAL ACCOUNT

To develop our account, we start with a paradigmatic human example (taken from: Newen, Griem, & Pika, 2022) to motivate our theoretical framework. We use this as basis to describe our multidimensional account and justify modified and more concrete dimensions.

*Tom lives with his old neighbour in Corona‐times, and she tells him that she suffers from isolation and that she is sad that she is no longer able to cook any complex meals but has to deal with simple cuisine due to her diminishing abilities. Since Tom knows her quite well, he is aware of the fact that she does not want any fancy meals that one could easily order from a restaurant, but she prefers a special type of cooking which has to be self‐made. Tom feels sorry for her and decides to cook and bring her favourite meal for her upcoming birthday. She is delighted*.

We argue that this case involves the characteristic components of full‐blown empathy, namely the *registration* of the other's relevant mental states or processes (‘state’ for short in the following) including the affective state. This is combined with the *attitude* to take care of the other and the *selection of a supporting action* that accounts for the mental states and the situation of the person. More precisely, we think that we can cluster relevant aspects into three main components: (*a*) the registration of the other's affective state (her sadness), of the situation she is in (her inability to cook) and also of her individual mindset (her desire to have the meal again); (*b*) an attitude to care for the other – an (at least implicit) desire to alter the other's state or situation; and (*c*) an other‐oriented response involving a self–other distinction, thereby trying to improve the other's situation and affective state (by bringing her favourite meal). In the case of human empathy, all these criteria can be easily investigated, especially relying on linguistic communication. In the case of nonhuman animals but also in the case of non‐linguistic infants, we need to substitute the features (*b*) and (*c*) such that all of them are systematically connected with behaviourally measurable cognitive abilities. To reach this aim, we start with a minimal condition and then we want to constrain it with testable cognitive abilities. The minimal condition is: *An agent* (A) *is registering another agent's* (B) *affective state in a certain way* x *and the behavioural reaction* r *of* A *is directly related to this registration and is realized in a certain way* y. If the minimal condition is satisfied, we have a candidate for empathy. To clarify whether it actually is a case of empathy we suggest a framework of five dimensions of cognitive abilities including three dimensions accounting for the relevant registration, namely (1) *registering the other's affective state*, (2) *registering the relevant situation* and (3) *registering the relevant mental states or mindset of the other* (cognitive perspective‐taking); furthermore, we need two further dimensions to characterize the relevant behavioural reaction, namely (4) *behavioural and cognitive flexibility* and (5) *social coordination*. These can substitute the two human‐centred dimensions, namely an attitude to care for others and the other‐directedness of the response behaviour. This comes with two advantages. Most importantly, we have a substitute for the cognitive ‘attitude to care for the other’ which is mainly inaccessible without linguistic exchange. And, we characterize social coordination such that we can include simple cases like emotional contagion which do not involve a self–other distinction. The dimension of flexibility has the additional role to exclude hard‐wired prosocial behaviour which need not (always) be triggered as a behavioural response related to the registration of the affective state of the other.

To develop this further, we provide a test case adapted from Bartal *et al*. (2011):

*There are two cage mate rats: one can freely move while the other is distressed because she is captured in a cage without food. The freely moving rat learned how to open the cage with a lever in a previous situation without any other rat being involved. Now she is hungry and notices some tasty chocolate easily in reach while also registering the distress of the cage mate rat. What does she do? Despite being hungry, the freely moving rat first frees the cage mate before aiming for the food and even shares the chocolate*.

Is this a case of full‐blown empathy and, if so, how can we systematically analyse this case to offer a convincing description? Are there differences between this and human empathy or are such differences completely non‐existent? Or is this behaviour simply triggered by some basic selfish desire for social contact? Before answering these questions, we need to look at the state of the art. On the one hand, there are general reviews supporting the line of reasoning that rats are empathetic and that they may even be a good model to investigate the neural implementation of empathy in mammals (Kim, Keum, & Shin, 2018; Meyza *et al*., 2017). Rats are reported to show overlaps of behavioural flexibility, similar behavioural dispositions (e.g. familiarity bias, sex bias with females showing more helping behaviour) as well as similar brain circuits compared to humans (Meyza *et al*., 2017). Furthermore, there are additional studies supporting the observation of prosocial behaviour in rats (Bartal *et al*., 2014; Sato *et al*., 2015). But then there are also studies arguing that the helping behaviour cannot be interpreted as a case of empathy because it is just a result of the desire for social interaction (Silberberg *et al*., 2014) such that if an opportunity for social interaction after freeing the cage mate is lacking, they refrain from prosocial helping behaviour (Heslin & Brown, 2021). But against this radical criticism speaks one study which demonstrates that rats display empathetic behaviour independent of the opportunity for social interaction (Cox & Reichel, 2020). Clearly, observing the helping behaviour of rats is quite impressive, but the interpretation of this behaviour remains a challenge. How can we make progress in evaluating this case? We think that even further studies of rats will not justify a claim that either rats are acting fully empathetically or completely non‐empathetically but that rats seem to realize a certain variant of empathy which is not yet full‐blown empathy as we can observe it in paradigmatic human cases. We need a new framework to describe and characterize variants of empathy and to integrate the rat case.

## III. THE SHORTCOMINGS OF TWO CENTRAL ACCOUNTS OF ANIMAL EMPATHY

Do we really have to develop a five‐dimensional space to account for empathy? Why can we not just rely on simpler approaches to animal empathy? One prominent model inspired by ontogeny is the Russian doll model suggested by de Waal & Preston (2017). They claim that empathy relies on a basic perception–action mechanism that enables emotional contagion. Further abilities like sympathetic concern and consolation rely on this foundation, and the latter are the basis for perspective‐taking and targeted helping. The sequence of systematically developing cognitive abilities should explain all cases of empathy. Thus, we receive a linear model of stepwise developing cognitive abilities enabling increasingly complex forms of empathy (see Fig. 1). The idea of a basic perception–action coupling with only minimal cognitive prerequisites (for a detailed description see Preston & de Waal, 2002) is an interesting idea for a mechanism enabling basic empathetic behaviour in a broad variety of species. They also show convincing evidence for the evolution of such a mechanism and how it is closely related to emotional contagion and forms of empathy inducing an emotional response in the observer.

**Fig. 1:**
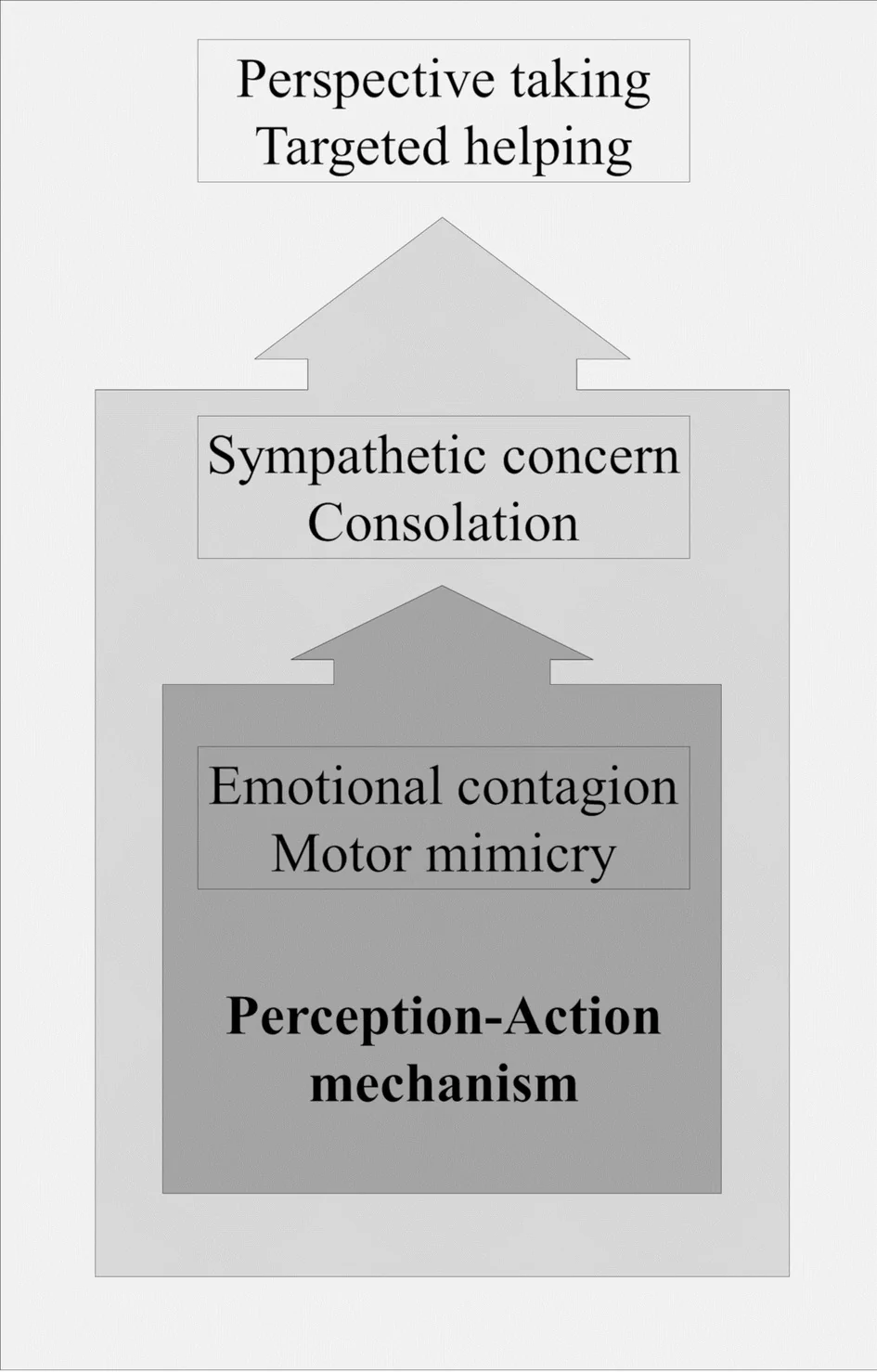
The Russian doll model (adapted from de Waal & Preston, 2017) showing levels of empathetic abilities.

Although we agree that the ontogenetic perspective is fruitful, the strict linearity presupposed in de Waal & Preston (2017) faces a challenge: it cannot account for several cases of pure cognitive empathy. For example, in the case of psychopathy or narcissistic personality disorders (NPD) the patients are very good in cognitive perspective‐taking and targeted helping, but this does not rely on sympathetic concern although the latter is supposed to be the basis of the former. Psychopaths thus can use their complex abilities to harm others due to lack of sympathetic concern (Veit *et al*., 2002; Verona *et al*., 2004). Preston *et al*. (2020) challenge this critique with a review in which they aim to demonstrate that cognitive empathy is always based on affective empathy. They describe that people with NPD are still able to activate affective empathy, just to lesser amounts or with less motivation. This evidence does not exclude that people with NPD seem often to realize cognitive empathy without (or almost without) affective empathy being involved although they remain able to activate affective empathy. Another analogous case of pure cognitive empathy is that of professional medical doctors dealing with patients: a professional anaesthetist is better off not being so sensitive (although they are able to be), not to trigger emotional contagion in the case of a fearful patient. Just encouraging the patient and calming them down could be the perfect professional treatment with an attitude of sympathy but lacking any emotional contagion. The case of cage freeing behaviour in rats is a challenge for the Russian doll model too, despite the open interpretation: if you take the rats' behaviour to be targeted helping (in line with Bartal *et al*., 2011), then the problem is that targeted helping and perspective taking should cluster together but so far we have almost no evidence that rats can realize perspective taking in terms of taking over the perspective of another individual. If you take the position that the freeing behaviour is only driven by the selfish desire for social interaction (in line with Heslin & Brown, 2021) or escape from aversive ultrasonic vocalizations, then it remains unclear why the freeing behaviour is systematically done first and then followed by the sharing of food. Thus, the Russian doll model seems to have problems to account for this complex rat behaviour. We admit that Preston *et al*. (2020, p. 347) start with the observation that “empathy is a complex, multi‐dimensional capacity”, but they still insist on the linearity of the Russian Doll model while we think that evolutionary plurality can be better accounted for in a more flexible, multidimensional profile account.

A second prominent account of animal empathy is the combination model (Yamamoto, 2017). It describes empathy as based on three typical *cognitive or behavioural abilities*: matching with others, understanding others and prosociality. It involves the claim that empathy can be described as involving at least one of these abilities while full‐blown cases of empathy involve all three abilities. While this model avoids the problem of strong linearity, it entails the claim that realizing one factor alone (e.g. prosocial behaviour) is sufficient to be categorized as a case of empathy. But this is implausible. Prosocial behaviour may be inborn behaviour, such as helping behaviour in ants, which is rigid and independent of registering any affective states of the other (Miler, Yahya, & Czarnoleski, 2017). Also, in humans we would not classify prosocial behaviour which is unrelated to the affective state of a person as empathetic; for example, if Tom brings the favourite meal to the old lady just because he was cooking it to prepare for a cooking competition and he was giving it to all the neighbours to ask them for their evaluation. If the relevant relation to the affective state of the other is lacking, prosocial behaviour might be evolutionarily important for a group but is not a case of empathetic behaviour. To avoid the shortcomings of these two models, we need an alternative and propose our multidimensional profile account.

## IV. TOWARDS A MULTIDIMENSIONAL AND MULTI‐FEATURE ACCOUNT OF EMPATHY

The starting point is the minimal condition (see Section II) which includes the registration of the other's affective state in a certain way and a systematically linked behavioural response of a certain type. We presuppose a minimal notion of *registration* as it is developed in Butterfill and Apperly (2013); thereby we include not only explicit but also implicit forms of sensitivity of relevant states and situations. To spell out our proposal, we suggest five dimensions of cognitive abilities which generate a multidimensional space to characterize varieties of empathetic behaviour. This tool can be used to analyse the cognitive situation and behaviour of a species but in principle also of a single individual. We focus on the former since we are aiming to develop a model of empathy for comparative research. These five dimensions typically involved in the realization of empathy are illustrated in Fig. 2. For a detailed investigation of the five dimensions, it must be clarified which kinds of observable and testable features indicate a realization of each of them. A detailed summary of all dimensions, the implementing underlying features and the fruitful candidates for experimental paradigms implementing each feature are described in Table 1.

**Fig. 2:**
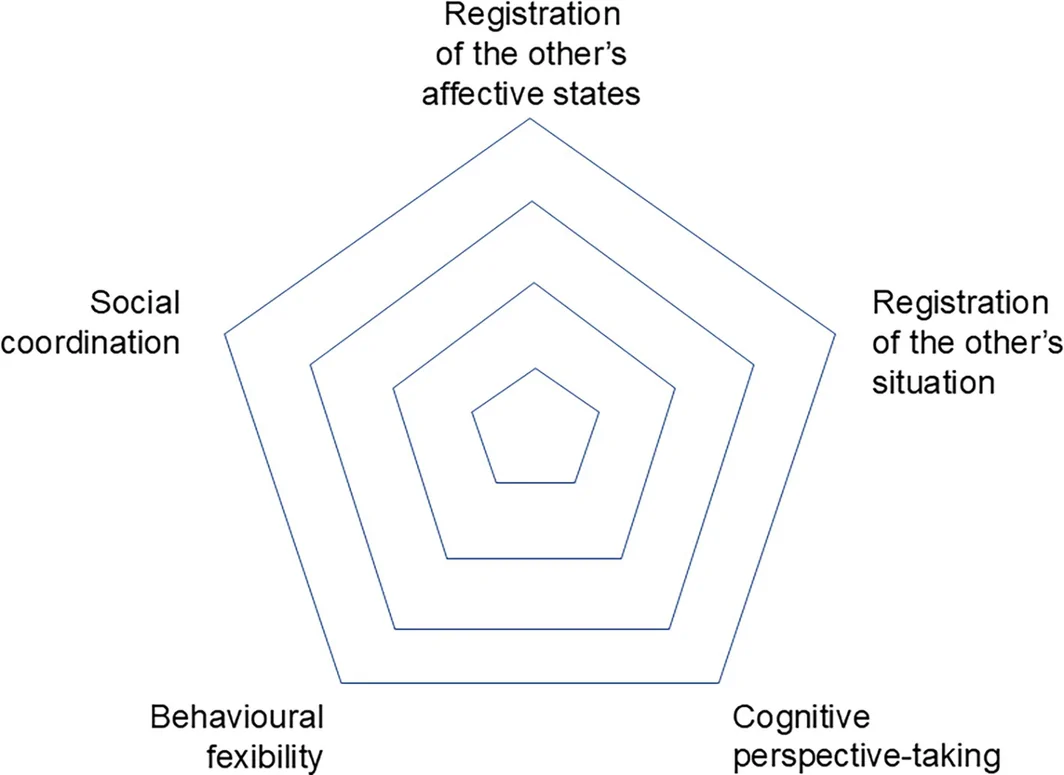
Dimensions of cognitive abilities involved in empathy as the basis for a species comparison.

**Table 1:** Five dimensions of empathy and their indicating features (cognitive abilities) as well as relevant test paradigms enabling empirical investigation.

Dimension	Feature	Paradigms
Registering the other's affective states	*Motor mimicry*	Contagious yawning; rapid facial mimicry; body posture & movement mimicking
	*Affective valence sensitivity*	Emotional contagion
	*Recognize basic emotions*	Adapt behaviour to basic emotions of others
	*Recognize complex emotions*	Adapt behaviour to complex emotions of others
Registering the other's situation	*Others as agents vs objects*	Biological motion‐detection
	*Others as goal‐directed actors*	Understanding goal‐directed actions; manipulation of others
	*Recognize relationships with and between other individuals*	Individual recognition; measure of behaviour & time spent for interaction; third‐party relationships
	*Others as members of a group with specific rules*	Rule understanding; third‐party punishment
	*Others as individuals with a personal past and future*	Individual recognition over longer time periods; what‐where‐when memory; planning & anticipating others' future behaviour
Cognitive perspective‐taking	*Visual perspective‐taking 1*	Gaze‐following behind barriers; guesser‐knower task
	*Preferences of others*	Hand over others preferred object
	*Attentional state of others*	Unwilling versus unable & similar paradigms
	*Visual perspective‐taking 2*	Know how others see something
	*Theory of mind*	False‐belief‐task
Behavioural flexibility	*Inhibitory control*	Stop signal task / cylinder task / similar tasks; delay of gratification task
	*Reversal learning*	Stop signal task with reversal of learned discrimination
	*Innovative problem solving*	Basic rule generalization; abstract rules; innovation
	*Means‐end reasoning*	Tool use, choice, manufacture
	*Social learning*	Model learning; true imitation
Social coordination	*Synchronization*	Automatic synchronization
	*Cooperation*	String pulling task; direct helping; taking different roles in a team/game/hunting; prisoner's dilemma
	*Consolation*	“Loser” consolation
	*Reciprocity*	Direct/indirect reciprocity
	*Prosociality*	Helping others without benefit/with costs; helping strangers with costs

The first dimension, registering the other's affective state, can also be characterized as bodily sensitivity. It typically involves four different features: (1*a*) the abilities of motor sensitivity to others' movements; (1*b*) perceiving affective valence of others' affective states; (1*c*) recognizing others' basic emotions; and (1*d*) understanding others' complex emotions. These features measure the registration of the other agent's affective state with increasing fine‐grainedness in line with ontogenetic classification of affective episodes (Newen *et al*., 2015).

For the second dimension, we suggest five different features which characterize different ways of recognizing the other in a situation: (2*a*) the ability to perceive others as agents as opposed to objects based on biological motion detection; (2*b*) to perceive others as goal‐directed actors (potentially including the capacity for joint attention and manipulability); (2*c*) to evaluate third‐party relationships; (2*d*) being sensitive to group rules and their application to others in a situation; and also (2*e*) perceiving others as individuals with a specific past and future.

The third dimension, cognitive perspective‐taking, can be described as understanding different features of others' mental states or processes. This can be measured by investigating the abilities of: (3*a*) visual perspective‐taking 1 (3*b*) visual perspective‐taking 2 (Flavell *et al*., 1981); (3*c*) registering the desires of others; as well as (3*d*) the ability to attribute beliefs and other propositional attitudes also called Theory‐of‐Mind ability.

The fourth dimension, behavioural flexibility, involves the degree of flexibility with which the species (or individual) can interact with its environment in a certain situation and across contexts. This is typically assessed by testing four different features: (4*a*) means–end reasoning as expressed in tool use and tool manufacture; (4*b*) reverse learning and problem solving; (4*c*) social learning; and (4*d*) inhibitory control.

Finally, social coordination is concerned with the degree to which other individuals are integrated into the behavioural schemata of a species (or individual). This other‐directedness can be assessed by measuring abilities of: (5*a*) synchronizing; (5*b*) cooperation; (5*c*) consolation; (5*d*) reciprocity; and (5*e*) magnitude of the costs individuals are willing to take for the benefit of others. The latter feature is connected to the aspect of altruism (Preston, 2013).

One may wonder why we do not add ‘self–other‐differentiation’ as an additional dimension since we agree with many researchers (Eklund & Meranius, 2021) that it is relevant for many cases of empathy. But the operationalizations of social coordination already involves different degrees of self–other differentiation (5*b* to 5*e*; only 5*a* synchronization does not presuppose it). Furthermore, it is involved in (almost) all features testing dimensions 1, 2 and 3. Thus, the measure of self–other differentiation is relevant but spread over four dimensions, and thus indicated by the degree of the realization of these dimensions. Due to lack of independence, we do not add it as its own dimension.

Now, we need to assess each of these dimensions and features on its own to generate a profile of empathy. We expect quite a variety of combinations of typical cognitive abilities in different species. The framework we propose uses five dimensions, records cognitive features for each dimension, and measures these on a rather coarse‐grained scale for indicating the level of performance for each feature. And, by aggregating scores, we also generate a measure of performance for each dimension. All three structural aspects of the profile characterization (the number of dimensions, the number of implementing features and the level of differentiation of the scales) are chosen pragmatically for our main explanatory purpose, namely to enable the most informative multi‐species comparison concerning empathy. We are open to adjusting the structural aspects of the framework for other explanatory purposes, e.g. for a more fine‐grained comparison of different age‐groups within one species.

To apply the framework, we need some technical aspects. The features of one dimension are not presupposed to hierarchically relate to any others such that one is presupposing the other. We leave the relation open and rather think of them as the relevant variety of abilities (including basic and complex abilities) that can be used to fruitfully indicate the level of performance of the relevant dimension. Technically we suggest the following evaluation process: each ability within a dimension can be either absent (0), weakly present (2) or strongly present (4). Depending on the presence or absence of all cognitive abilities in one dimension, this dimension itself gets a rating ranging from absent (0) to intense (4), with the intermediate stages minimal (1), medium (2) and high (3). This rating is calculated by summing the ratings of the abilities and dividing them by the number of abilities.

## V. SYSTEMATIC APPLICATIONS TO COMPARE NONHUMAN ANIMALS

To apply the multidimensional profile account to a species comparison, we start with a collection of the relevant evidence for each species concerning each dimension and each feature to generate a species‐specific profile of empathy. These profiles might then be compared to each other either in total or concerning one of the relevant dimensions. We start using our framework with a comparative description of humans and rodents. For these two taxa we describe each dimension and the relevant features in detail as a basis for a comparison (Sections (1), (5)). We then widen this comparative perspective by including great apes, corvids, and canids and highlight important comparisons between the different families (Section V.6). For these additional families the relevant features and empirical evidence of their performance is presented in Table 2. This enables us to offer a fruitful comparison of species‐sensitive profiles of empathy. Let us make explicit that there are two types of attitudes to attributing cognitive abilities to nonhuman animals: the *animal ethics perspective* which aims to prevent harm as much as possible and thus is generous in interpreting animal behaviour as indicative of cognitive abilities, and the *animal cognition perspective* which is more parsimonious to avoid anthropomorphism. We set priority to the animal cognition perspective in this article. We also think that this approach may set minimal standards for animal ethics.

**Table 2:** Dimensions of empathy and features that are realised in different species. While we summed different species in brackets under broader categories, empirical evidence is still different between these species in some cases. For this first analysis, we used the highest number for which evidence in any of the investigated species of the group was convincing. The first number (P) indicates the degree of presence of an ability: 0 = absent; 2 = weakly present; 4 = strongly present. The second number (R) indicates the level of reliability: 0 = no findings (and cases of very weak evidence); 1 = discussed / suggestive; 2 = commonly accepted / convincing.

Dimension	Feature	Rodents (*rats & mice*)	Great Apes (*chimpanzees, bonobos, & orangutans*)	Canids (*dogs & wolves*)	Corvids (*ravens, New Caledonian crows, & jays*)
Registering the other's affective states	*Motor mimicry*	Moyaho *et al*. (2015); Yu *et al*. (2017) **P2.R2**	Anderson *et al*. (2004); Fuhrmann *et al*. (2014); Davila Ross *et al*. (2008) **P4.R2**	Romero *et al*. (2014); Buttner & Strasser (2014); Palagi & Cordoni (2020); **P4.R2**	Wenig *et al*. (2022); Loretto *et al*. (2020) **P2.R1**
	*Affective valence sensitivity*	Meyza *et al*. (2017); Pellis & McKenna (1992); Hernandez‐Lallement *et al*. (2020) **P4.R2**	Mitani & Gros‐Louis (1998); Whiten *et al*. (2001); Parr & Hopkins (2000) **P4.R2**	Palagi *et al*. (2015); Quervel‐Chaumette *et al*. (2016); Huber *et al*. (2017) **P4.R2**	Wenig *et al*. (2021); Adriaense *et al*. (2019); Hahn *et al*. (2026) **P4.R2**
	*Recognize basic emotions*	Meyza *et al*. (2017); Smith *et al*. (2021) **P2.R2**	Parr (2001); Heesen *et al*. (2024); Nieuwburg *et al*. (2021) **P4.R2**	Albuquerque *et al*. (2016); Müller *et al*. (2015); Albuquerque & Resende (2023) **P4.R2**	Marzluff *et al*. (2012); Hooper *et al*. (2021) **P4.R2**
	*Recognize complex emotions*	Meyza *et al*. (2017) **P0.R1**	Boesch & Boesch‐Achermann (2000); de Waal (2008) **P0.R1**	**P0.R0**	**P0.R0**
Registering the other's situation	*Others as agents vs objects*	MacKinnon *et al*. (2010) **P2.R1**	Hare *et al*. (2000); Call *et al*. (2004); Tomonaga *et al*. (2004); [Gorillas: Gómez (1990); Prieur & Pika (2020)] **P4.R2**	Völter & Huber (2022); Abdai *et al*. (2021); Boch *et al*. (2023) **P4.R1**	Kondo *et al*. (2012); Bird & Emery (2008) **P4.R2**
	*Others as goal‐directed actors*	Reinhold *et al*. (2019) **P2.R1**	Call *et al*. (2004); Melis *et al*. (2006); Melis *et al*. (2011) **P4.R2**	Marshall‐Pescini *et al*. (2014); Völter *et al*. (2023); Gedai *et al*. (2026) **P4.R2**	Bugnyar & Heinrich (2006); Bugnyar & Kotrschal (2004); Miller *et al*. (2025) **P4.R2**
	*Recognize relationships with and between other individuals*	Moura‐Conlon *et al*. (2010) **P0.R1**	Goodall (1986); Watts (2006); Wittig & Boesch (2005); Mielke *et al*. (2017) **P4.R2**	Silver *et al*. (2021); Chijiiwa *et al*. (2022); Anderson *et al*. (2017) **P4.R1**	Massen *et al*. (2014); Paz‐y‐Miño *et al*. (2004); Hahn *et al*. (2025) **P4.R2**
	*Others as members of a group with specific rules*	**P0.R0**	Whiten *et al*. (2005) (one single study so far); territory papers: Wilson *et al*. (2001); Mitani *et al*. (2010) **P4.R2**	**P0.R0**	Boucherie *et al*. (2022); Chiarati *et al*. (2010); Kings *et al*. (2023); Arbon *et al*. (2025) **P4.R2**
	*Others as individuals with a past and future*	Moura‐Conlon *et al*. (2010) **P2.R2**	Wittig *et al*. (2014); Mitani & Watts (2001); Janmaat *et al*. (2014); anecdotal evidence: de Waal (2009); **P4.R1**	Fugazza *et al*. (2016) **P2.R1**	Müller *et al*. (2017); Boeckle & Bugnyar (2012); Kings *et al*. (2023) **P4.R2**
Cognitive perspective‐taking	*Visual perspective‐taking 1*	Reinhold *et al*. (2019) **P2.R1**	Hare *et al*. (2000); Bräuer *et al*. (2007); Kaminski *et al*. (2008) **P4.R2**	Kaminski *et al*. (2009); Maginnity & Grace (2014); Catala *et al*. (2017) **P4.R2**	Davidson *et al*. (2015); von Bayern & Emery (2009); Bugnyar *et al*. (2004) **P4.R2**
	*Registering other's preferences*	**P0.R0**	Yamamoto *et al*. (2012); Melis & Tomasello, (2013) **P4.R2**	Turcsán *et al*. (2015); Piotti & Kaminski (2016) **P4.R1**	Ostojić *et al*. (2014); Ostojić *et al*. (2013) **P4.R2**
	*Attentional state of others*	**P0.R0**	Liebal *et al*. (2004a); Liebal *et al*. (2004b) **P4.R2**	Völter *et al*. (2023); Schünemann *et al*. (2021); Marshall‐Pescini *et al*. (2014); **P2.R1**	von Bayern & Emery (2009); Dally *et al*. (2006); Bugnyar & Heinrich (2005) **P4.R2**
	*Visual perspective‐taking 2*	**P0.R0**	Karg *et al*. (2016) **P0.R1**	Huber *et al*. (2025) **P2.R1**	Bugnyar *et al*. (2016) **P2.R1**
	*Theory of mind*	**P0.R0**	Krupenye *et al*. (2016); Kano *et al*. (2017), but see: Heyes (2017); Krupenye *et al*. (2017) **P2.R1**	Lonardo *et al*. (2021); Lonardo *et al*. (2025) **P2.R1**	**P0.R0**
Behavioural flexibility	*Inhibitory control*	Mayse *et al*. (2014) **P4.R2**	Völter *et al*. (2022); Herrmann *et al*. (2007); Amici *et al*. (2008) **P4.R2**	Brucks *et al*. (2017); Müller *et al*. (2016) **P4.R2**	Kabadayi *et al*. (2016); Hillemann *et al*. (2014); Dufour *et al*. (2012) **P4.R2**
	*Reversal learning*	McAlonan & Brown (2003) **P4.R2**	Schusterman (1964); Wobber *et al*. (2010); Rosati (2025) **P4.R2**	Van Bourg *et al*. (2021); Piotti *et al*. (2018); Wallis *et al*. (2014) **P4.R2**	Bond *et al*. (2007) **P4.R1**
	*Innovative problem solving*	Neves Filho *et al*. (2015); Blaisdell *et al*. (2006) ***P4.R2***	Goodall (1986); Herrmann *et al*. (2007); Pika *et al*. (2019) **P4.R2**	Lazarowski *et al*. (2020); Range *et al*. (2011); Aust *et al*. (2008) **P4.R2**	von Bayern *et al*. (2009); Auersperg *et al*. (2011); Heinrich & Bugnyar (2005) **P4.R2**
	*Tool use & manufacture*	Nagano & Aoyama (2017) **P2.R2**	Köhler (1924); Goodall (1986); Boesch & Boesch (1982); Whiten *et al*. (1999) **P4.R2**	**P0.R0**	Jacobs & Osvath (2022); Gruber *et al*. (2019); Bird & Emery (2009); Pendergraft *et al*. (2023); Moll *et al*. (2025) **P4.R2**
	*Social learning*	Heyes *et al*. (1992) but see: Mitchell *et al*. (1999); Rautio *et al*. (2023); Yamada & Sakurai (2022) **P2.R2**	Marshall‐Pescini & Whiten (2008); van Leeuwen *et al*. (2014); Padberg *et al*. (2025); but see Neadle *et al*. (2020) **P4.R2**	Huber *et al*. (2018); Fugazza & Miklosi (2014); Pongrácz & Dobos (2025) **P4.R2**	Lee *et al*. (2019); Kulahci *et al*. (2016); Cornell *et al*. (2011) **P4.R2**
Social coordination	*Synchronization*	Heyes *et al*. (1992) but see Mitchell *et al*. (1999); Campbell & Heyes (2002); Casarrubea *et al*. (2024) **P2.R1**	Hattori *et al*. (2013); Hirata & Fuwa (2007); Schweinfurth *et al*. (2022) **P4.R2**	Duranton & Gaunet (2015); Wanser *et al*. (2021); Lamontagne & Gaunet (2024) **P4.R2**	Ling *et al*. (2019) **P4.R2**
	*Cooperation*	Łopuch & Popik (2011); Paulsson & Taborsky (2022); Wood *et al*. (2016) **P2.R2**	Mitani (2009); Samuni *et al*. (2021); but see Melis *et al*. (2016) **P4.R2**	Range *et al*. (2019); Marshall‐Pescini *et al*. (2017); Martínez *et al*. (2023) **P4.R2**	Asakawa‐Haas *et al*. (2016); Massen *et al*. (2015); Wascher & Bugnyar (2013) **P4.R1**
	*Consolation*	Luo *et al*. (2020); Du *et al*. (2020); [other rodents: Burkett *et al*. (2016)] **P4.R1**	Webb *et al*. (2017); Palagi *et al*. (2004); Cordoni *et al*. (2006) **P4.R2**	Cordoni & Palagi (2008); Cools *et al*. (2008); Cavalli *et al*. (2016) **P2.R1**	Sima *et al*. (2017); Fraser & Bugnyar (2010); Seed *et al*. (2007) **P4.R2**
	*Reciprocity*	Kettler *et al*. (2021), *Wood et al*. (2016); Mason (2021) **P2.R2**	Newton‐Fisher & Lee (2011); Watts & Mitani (2002); Mitani (2006) **P4.R2**	Gfrerer & Taborsky (2017); Gfrerer & Taborsky (2018); but see McGetrick *et al*. (2021) **P2.R1**	Fraser & Bugnyar (2012); but see: Wascher *et al*. (2020) **P2.R1**
	*Prosociality*	Bartal *et al*. (2011); Han, S., Chen, Y.‐Q., Zheng, B., Wang, Y.‐X., & Yin, B., in preparation; But see Heslin & Brown (2021) **P2.R1**	Van Leeuwen *et al*. (2021); Boesch *et al*. (2010); Mascaro *et al*. (2022) **P4.R2**	Van Bourg *et al*. (2020); Sanford *et al*. (2018); Dale *et al*. (2019) **P4.R1**	Horn *et al*. (2020); Massen *et al*. (2020); Horn *et al*. (2016); Schwab *et al*. (2012) **P2.R1**

### (1) Registering the other's affective state

The first ability on the dimension of registering the other's affective state is *motor mimicry*. In humans, mimicry is well established. Mothers tend to open their mouth in response to infants opening their mouths when they are fed (O'Toole & Dubin, 1968). Chartrand & Bargh (1999) examined facial mimicry in laboratory experiments and found that individuals interacting with a smiling confederate smiled more than those interacting with a confederate who did not smile. More generally, they demonstrated that mimicry occurs automatically in standard dyadic interactions. Similar behaviours are found in rats and mice but only proven for contagious scratching and yawning (Moyaho *et al*., 2015; Yu *et al*., 2017). However, the observation of motor mimicry only indicates a sensitivity to others' motoric activities, but it remains open whether it is a sensitivity to others' affective states. Thus, mimicry has a dual role: we consider it to be illustrative of the lower bound, not yet being a case of empathy, but we also think that it is an ability that typically clusters with other abilities related to being sensitive to others' affective states, and thus include it as an indicating feature. Summarizing the motoric sensitivity, it is advanced in humans, however there is no evidence that rats show similar sensitivity for body posture and movements of others. Thus, humans are graded as having a strong presence (4) of the feature motor mimicry, while rats were graded as 2 (weakly present) due to a lack of evidence.

The second feature we evaluate here is a sensitivity for the *affective valence* of the emotions of others. The most common approach for assessing this ability is to test emotional contagion. The newborn reactive cry is one prime example of how infants are directly influenced by the emotional state of others, as they take positive or negative valence (Geangu *et al*., 2010). Rats also show emotional contagion for positive and negative valences. Examples are affective contagion of distress (Carrillo *et al*., 2019; negative valence: Meyza *et al*., 2017) and that of playfulness (positive valence: Pellis & McKenna, 1992). So, both, humans and rats appear to be highly sensitive to the affective valence of others, yielding a strongly present (4) attribution for this ability.

The next feature is the recognition of *basic emotions* of others. Happiness, sadness, fear, disgust, anger, and surprise are often considered as basic, since they appear to be present in all human cultures from early on in infancy (Ekman, 1992), as well as in many other mammalian species (Bekoff, 2000). Although the concept of basic emotions is debated (Ortony & Turner, 1990), it is successfully used as a basis for research, especially in animal research (Scarantino & Griffiths, 2011). Humans can distinguish types of basic emotions reliably and easily in explicit recognition tasks of face‐based recognition of emotions (Ekman, 1992) or implicitly in daily social communication (4, strongly present). Rats apparently also adapt their behaviour according to their mates' basic emotional signals, concerning at least happiness, disgust, and fear (Meyza *et al*., 2017). Whether they can react in a similar fashion to sadness, anger or surprise remains unclear due to a lack of data. So, they possess the ability to implicitly recognize some of others' basic emotions (2, present), while further research would be necessary to provide evidence for a strong presence of this feature.

Recognizing *complex or cognitive emotions* (Zinck & Newen, 2008) of others is the fourth feature that we suggest within this dimension. Complex emotions include some cognitive appraisal of the situation which enables humans to experience guilt, shame, or jealousy. Apparently, children must learn to not only recognize these emotions in others, but also to express them. Some of them are acquired earlier (the first signs of shame are shown at the age of two: Rochat & Striano, 2002), others later in development. Although there seems to be quite a variability in recognizing complex emotions dependent on age, cultural similarity and educational background, humans on average are highly sensitive to complex emotions (4, strongly present). Whether rats possess the ability to experience complex emotions is highly debatable, depending on the kind of emotion tested as well as on the chosen explanation for a shown behaviour (see Table 2; Meyza *et al*., 2017). From a critical perspective, the reports of registering complex emotions are not grounded in any convincing study. Thus, we evaluate this as completely missing (0, not present).

Let us summarize the evaluation of the dimension of registering the other's affective state using the proposed calculation: Humans reached a 4 for all four features and thus in average receive a 4 as the value for this dimension [equation: sum (4 + 4 + 4 + 4)/4[Number of features] = 4]. Rats only receive a 2, indicating a medium performance level abilities in this dimension [(2 + 4 + 2 + 0)/4 = 2]. Now we can proceed analogously with the remaining dimensions to reach a comparative profile.

### (2) Registering the other's situation

Registering the relevant situation of the other requires that an organism can evaluate the relevant situation, context or specific circumstances. The basic ability consists of *distinguishing others as* (*animate*) *agents from* (*inanimate*) *objects in the environment*. This can be achieved by several mechanisms, one of which is biological motion‐detection. Biological motion detection is evaluated as a hallmark of social cognition (Pavlova, 2012) which is underlined by the fact that severe deficits are specifically correlated with high‐functioning autism (Congiu, Schlottmann, & Ray, 2010). Most relevant here is that it is also an evolutionarily old capacity. It is clearly shown to be present in cats (*felis catus*) (Blake, 1993). Concerning rats, there is some evidence that they are capable of distinguishing movement patterns when seeing light dots (MacKinnon, Troje, & Dringenberg, 2010) but evidence for a strong presence of this ability is lacking. For these reasons, rats obtain a (2) while humans receive a (4).

Another ability in this dimension is that of perceiving *others as goal‐directed actors*. This ranges from the perception of behavioural goals to more complex evaluations. One indicator is the ability of joint attention, and its initiation is observed in infants between the age of 9–14 months (Tomasello, 1999) or even earlier in development (Csibra, 2008). Already during the first year of life children learn to register goal‐directed actions of others: when children observe others' grasping acts, they learn to expect them to grasp for a certain goal object (Woodward, 1998). Further steps in understanding others' goal‐directed activities are described by the *teleological* account of social understanding in infancy (Perner, Priewasser, & Roessler, 2018). Fully developed understanding of others' goal directed actions can be observed in using others' as means for one's own goals. Accordingly, humans' ability to perceive others as goal‐directed actors is strongly present (4).

For rats this ability has not been deeply investigated. Reinhold *et al*., (2019) showed that rats perform interesting deceit‐like behaviours in a hide‐and‐seek game with their human experimenters, such as staying quiet while hiding as opposed to rather noisy seeking. This suggested that rats can register the human as a goal‐directed actor, although this may be constrained to situations involving themselves. However, further research is needed to confirm that this ability is reliably shown by rats across different situations. Thus, they stay with (2), weakly present. This might have to be changed with further evidence.

Another interesting ability of social animals is that of *recognizing relationships with other individuals and between other individuals*. Basic characteristics of a relationship might be rank, kinship, time spent together, friendliness, and others. This can be manifested in a dyadic manner, when an animal recognizes its relationship to a species member, or in a triadic manner, when the relation between two other individuals is assessed and potentially tracked over time according to events that might influence the relationships, such as rank fights. Humans are particularly developed in this kind of social skill. We manage a great variety of highly dynamic relationships that change over time. Further, we can evaluate third‐party relationships on a concrete (e.g. between two of our friends) as well as an abstract level (e.g. the relationship of police officers of different ranks in general). Thus, humans receive a (4) for this ability. Some evidence suggests that rats might also be capable of maintaining different kinds of relationships with other individuals, as they have a long‐term memory for individual recognition (Moura‐Conlon, Meirelles, & Xavier, 2010). Whether they can evaluate third‐party relationships and, if so, to which degree, has not been a topic of investigation, and data are scarce and debatable on this topic in general. Thus, rats again gain a (0) here, since the ability to recognize relationships in general lacks convincing evidence.

In addition to personal relationships between individuals, others can be seen as *members of a group with specific rules*. At the basis of this minimal notion of rules is a general sensitivity for learned rules that apply also to others. This could be shown by behavioural reactions to others breaking the rules, such as punishment or other reprimanding measures. Humans live in huge societies with explicit laws and implicit social rules. As early as the age of three children are already sensitive to rules and tend to teach rules to others and even advise (either implicitly or explicitly) to follow the rules (Köymen *et al*., 2015; Rakoczy, 2008). Thus, rule understanding and the application of these rules to other ingroup members is strongly present (4) in humans. While rats learn rules quickly and can apply them to similar circumstances (Murphy, Mondragón, & Murphy, 2008), there is no evidence for reprimanding behaviour to conspecifics breaking the rules, apart from punishment by dominant individuals. So, up to now, a perception of others as members of a certain group with specific rules to obey seems to be mostly absent (0) in rats. Obviously, further studies could yield an insight into rats' social interactions and perception of third‐party conflicts.

The last interesting feature is perceiving *others as individuals with a personal past and future*. The most basic form would be recognizing conspecifics as the same individuals on different occasions. Both, humans and rats (Moura‐Conlon *et al*., 2010), can do that. A more sophisticated example is anticipating future behaviour of others beyond the current situation. Children learn this in parallel to thinking about their own past and future, while developing episodic memory around the age of three (Hayne & Imuta, 2011; Payne *et al*., 2015). While there is evidence for episodic‐like memory in rats (Crystal & Smith, 2014), anticipating future behaviour of others has not been shown. Thus, humans are rated with (4) again, while rats only gain a (2).

Summing up, humans, again, reach a 4, while rats only come as far as 1.2 [(2 + 2 + 0 + 0 + 2)/5 = 1.2] in the realm of understanding others as individuals in a specific situation.

### (3) Cognitive perspective‐taking: registering the other's cognitive states

In addition to registering the other's affective state and situation, we can receive an even more detailed picture of the other's actual mindset if we can rely on the ability of cognitive perspective‐taking. This involves the registration of others' mental states such as perceptual states, knowledge, beliefs, desires, general preferences, etc. We can operationalize this dimension with five features. The first two features consist of the two types of *visual perspective‐taking* (VPT): the ability to register *what* others can and cannot see (VPT 1): “I can see a banana that is not visible for the other”; and *how* others perceive an object by accounting for perspective‐variant features (VPT 2): “the other perceives a car from the back and I perceive it from the front”. VPT 1 develops in human infants from 14 months onwards (Sodian, Thoermer, & Metz, 2007), while VPT 2 has been shown earliest in three‐year‐olds (Moll & Meltzoff, 2011). Humans appear to be extraordinarily successful in using these abilities during social interactions, placing them at a (4) for both features in question.

Rats' behaviour in the hide‐and‐seek game (Reinhold *et al*. 2019) provides interesting evidence that rats can engage in VPT 1. They only stay silent when they are themselves hiding and not while seeking, thereby indicating that they have a sensitivity for what others can perceive. However, there is no evidence suggesting that VPT 1 in rats can be directed at a third entity, which would indicate a sensitivity to which objects others can see compared to themselves. So, rats gain a (2) for VPT 1. There is no evidence that rats can engage in VPT 2, so we must leave them at (0) for now.

Registering the *attentional states* of others is another interesting ability in this dimension. Basic tests for attentional sensitivity involve gaze‐following, more complex forms are tested by ‘unwilling’ *versus* ‘unable’ tasks. Advanced forms involve full‐blown joint attention which develops very early in human children. So far, rats have not been shown to pass any of these. Thus, we rate humans with (4), while rats are left at (0).

Behaving in the light of *preferences of others*, even when they are different from one's own, is the fourth feature of cognitive perspective‐taking. Around age two children are already able to recognize others' preferences if they are similar to their own (Fawcett & Markson, 2010). Only from age three onwards they start to evaluate others' preferences correctly even if those are different from their own (Kushnir, Xu, & Wellman, 2008). Again, there is no evidence yet for rats. Therefore, humans again score (4), rats (0).

The fifth feature in the dimension of cognitive perspective‐taking is the ability to attribute (either implicit or explicit) beliefs and desires to others. This epistemic *theory of mind* (ToM) ability is usually tested via the false belief task (for a description of the explicit version see Wimmer & Perner, 1983). Children pass this explicit version at around age four, while they pass implicit versions at much earlier ages depending on the paradigm used (e.g. active helping at 18 months: Buttelmann, Carpenter, & Tomasello, 2009; violation of expectation at 15 months: Onishi & Baillargeon, 2005; for a systematic overview see de Bruin & Newen, 2012). There is a major debate concerning the replication crisis of implicit false belief tasks (Kulke *et al*., 2018) and about the question how implicit and explicit versions of the false belief task are related to each other given that some implicit versions still are replicable (Setoh, Scott, & Baillargeon, 2016). Rats never passed any of these tests so far; Although implicit versions can be used to assess ToM abilities in non‐linguistic species, there is so far only minimal evidence for nonhuman animals (apes: Buttelmann *et al*., 2017; Krupenye *et al*., 2016; dogs: Huber & Lonardo, 2023; Lonardo *et al*., 2021). Thus, we rate humans with (4) and rats with (0) for this ability.

Summing up, humans are extremely skilled in cognitive perspective‐taking (4), while there is so far only minimal evidence for cognitive perspective‐taking in rats [(2 + 0 + 0 + 0 + 0)/5 = 0.4].

### (4) Behavioural flexibility

The dimension of behavioural flexibility is introduced to exclude inborn behavioural reactions which seem to be empathic but do not deserve to be classified as such. We start with two features to test behavioural flexibility, namely *inhibitory control* and *reversal learning*. Although both abilities are interdependent, we have two rather different types of tests of inhibition‐based behaviour and thus take them to be two features indicating flexibility. Rats pass the stop signal task, showing proactive and reactive inhibitory control (Mayse *et al*., 2014), and have similar brain circuits involved in reversal learning as is shown in humans (McAlonan & Brown, 2003). Toddlers from 15 months onwards are capable of basic reversal learning, developing further with age (de Sousa, Gil, & McIlvane, 2015). Inhibitory control also shows a strong correlation with age and an unfolding during ontogeny (Carver, Livesey, & Charles, 2001). Thus, we evaluate both, rats and humans with (4) to appreciate their performance concerning these two features.

A third feature is *innovative problem solving*. This refers to the ability to generalize learned solutions and transfer them to novel problems as well as innovative approaches to solving a novel task. Recent evidence shows that preschoolers can understand abstract causal relations, hinting at a general ability to generalize causal effects across tasks (Goddu, Lombrozo, & Gopnik, 2020). However, only around age eight do they reliably solve the so called ‘Aesop's fable task’ in which they learn that dropping stones in water raises the water level and thus a desired object (a floating worm) will come into their reach (Cheke, Loissel, & Clayton, 2012). Obviously, humans are also capable of finding innovative solutions to various kinds of different problems, even though we might use simple heuristics in most everyday encounters instead of thinking them through. Rats also can not only solve problems by trial‐and‐error learning, but also apparently possess the ability to generalize across different physical tasks with similar causal mechanisms (Blaisdell *et al*., 2006). Furthermore, since there is now early evidence for innovative problem solving in laboratory rats (Neves Filho *et al*., 2015), both, humans and rats receive a (4) for problem solving skills.

A fourth feature is *means–end reasoning* as part of general cognitive flexibility. It is typically indicated by tool use and tool construction, as this involves an understanding of how the tool can be used to reach a goal (see Papineau, 2003). This is not applicable to species that are physically restricted from using tools efficiently but for rats there are some possibilities of tool use. It is common knowledge that humans use a variety of tools ranging in complexity from hammers to computers and that they are skilled in creating new tools for distinctive purposes. Being bipedal with hands free to grab physical objects and wield them with high dexterity renders us extraordinarily skilled in using and manufacturing tools (4). Rats are also able to use tools and choose them according to task‐relevant features (Nagano & Aoyama, 2017), but there is no evidence of tool construction or use of highly innovative tools. Thus, the ability to use tools is present (2), but only at an intermediate level.

The last feature that indicates behavioural flexibility is the *ability of social learning* understood in a wide sense, ranging from mere trial‐and‐error learning enhancements from observing others, to true imitation. Institutions like schools and universities, as well as our ability to truly imitate others' behaviours from rather early childhood onwards (Gergely, Bekkering, & Király, 2002) prove humans' specialty in this field (4). Rats are not easy to evaluate concerning this ability: one study reported that rats learn by observation to copy a behaviour with a joystick movement (Heyes, Dawson, & Nokes, 1992); but recent methodological criticism demonstrated that this impressive behaviour is based on low‐level sensitivity for scent and does not rely on observing the other agent's activity (Mitchell *et al*., 1999). Given this convincing parsimonious reinterpretation, we need to find studies excluding any odour guidance to support the claim that we are dealing with social learning. There are recently two such studies. First, one in which the observer rat can see another rat moving in a Barnes circular maze (Yamada & Sakurai, 2022). It was shown that the observer rats could speed up their learning by observing the behaviour of a knowledgeable model rat. After observing the model rat escaping into the goal box in the maze, the observer rats demonstrated significantly faster escape behaviour than the model rats. Since the metal platform on which the rats were running was cleaned after each performance, an orientation based on scent can be excluded. A second study (Rautio *et al*., 2023) demanded that the rats touched two objects in a field in the right sequence to receive a reward. The task was structured like a Pavlovian conditioning paradigm: the observer rats viewed model rats who tap two internally lit spheres in a particular sequence and, whenever the model rat performed a trial correctly, both animals received a reward in the form of a neural stimulation in the medial forebrain bundle. Again, observer animals outperformed control animals measuring task performance and efficiency. This performance cannot be based on sensitivity for scent since the model rat was tapping at both objects and the odour cannot reveal the sequence of tapping. Thus, we take these two experiments to be convincing evidence for an intermediate level of social learning in rats (2) compared to the sophisticated level in humans (4).

In sum, the evaluation of the different abilities results in a 4 for behavioural flexibility in humans and a remarkable 3.2 [(4 + 4 + 4 + 2 + 2)/5 = 3.2] in rats.

### (5) Social coordination

The final dimension is social coordination, i.e. to what extent and in which way are other agents considered and integrated into guiding one's own behaviour. In non‐basic cases this involves a self–other distinction which is involved in all cases of empathy going beyond emotional contagion (as highlighted by Lamm & Tomova, 2018). The first basic feature of this dimension is the ability of *synchronization* in the sense of attuning oneself to the behaviour of another individual by copying their behaviour. A prime example of basic synchronization is swarm behaviour of birds, insects or fish. However, humans are also tuned to what others do and often synchronize with others unconsciously during a conversation in tone of voice, speech rhythm and velocity, gestures, body posture etc., as synchronization elicits positive feelings towards the conversation partner (Clark, 1996; Louwerse *et al*., 2012) and even increases altruism (Valdesolo & DeSteno, 2011). Furthermore, humans can willingly coordinate their movements with others, as can be done in complex ways e.g. in synchronized swimming. Rats seem to be able to develop motoric coordination which is visible in motoric imitation behaviour (Heyes *et al*., 1992). Although their results are highly debatable (see Campbell & Heyes, 2002; Mitchell *et al*., 1999), there has been recent suggestive evidence for motor imitation in rats, using a more naturalistic task (Casarrubea *et al*., 2024). For now, it makes sense to evaluate humans with (4) and rats with (2) in this domain.

Another important feature of this dimension is cooperation. This ranges from cooperating in a simple task, such as pulling a string together, to taking different roles in a joint activity, e.g. in a football team, or during collaborative hunting. One of the first cooperative behaviours developing in infants is directed helping (Giner Torréns & Kärtner, 2017). From early on, humans engage in (semi‐)cooperative actions and games. Humans can also cooperate over longer periods of time including breaks and over generations to finish complex projects. All in all, humans can be extremely cooperative (4), although this ability might just as well be used to accomplish destructive goals. Rats can learn to cooperatively poke targets simultaneously to receive food (Łopuch & Popik, 2011) and succeed in a modified version of the prisoner's dilemma task (Wood, Kim, & Li, 2016). However, there are currently no signs of taking on different roles in collaborative tasks in rats or other rodents. Yet, evidence on other forms of cooperation is convincing, gaining rats a (2) for this feature.

A third behavioural ability often considered in the context of nonhuman empathy is *consolation*. Consolation in a broad sense encompasses all comforting behaviours offered to another individual in distress. Since the consoling contact reduces stress not only in the consoled but also in the consoler, this raises the danger of rating a behaviour as other‐oriented when it may be primarily self‐directed and only other‐directed in a derived sense. Although there remains some ambiguity, we take consolation behaviour to be a useful indicator of social coordination. Concerning humans, we observe consolation behaviour in preschoolers (Farver & Branstetter, 1994) and toddlers aged 18 to 25 months. They show it even when a victim of some harm does not express the negative emotion (Vaish, Carpenter, & Tomasello, 2009). Concerning rats there is good evidence showing consolation behaviour directed towards cage mates and strangers (Du *et al*., 2020; Luo *et al*., 2020; for other rodents see Burkett *et al*., 2016). Since their consolation behaviour is biased by the observer's past experience with pain and familiarity between the rats, this cannot simply be interpreted as merely avoiding an annoying squeak. So, both rats and humans, are rated with (4).

A fourth feature is the ability for *reciprocal actions* within a species. There are different approaches to distinguish between different kinds of reciprocity (for an overview on underlying mechanisms and evolutionary background see Nowak & Sigmund, 2005; Schweinfurth & Call, 2019). One approach focusses on the epistemic dimension, i.e. which sources of information about the other are registered and used to shape reciprocal behaviour. Generalized reciprocity (Pfeiffer, *et al*., 2005) only relies on simple heuristics of how often other individuals in general behave prosocially directed towards me and whether this registration influences me being prosocial to others. It does not include any bookkeeping of individual activities. The latter is characteristic for direct reciprocity which consists in mutual exchange of support or goods. One example for this are tit‐for‐tat strategies, i.e. you groom me, thus, I groom you. This mutual exchange can be even more sophisticated if it involves third‐party observations. More precisely, if I observe you engage in successful tit‐for‐tat exchanges with others regularly, I will be more disposed to act reciprocally towards you. Another interesting approach to reciprocity considers the structure of the reciprocal exchange itself. It can be direct, i.e. a steady back and forth between two partners, or indirect, i.e. a circular exchange between three or more individuals in the form of A helps B, B helps C, and C helps A, or open ended. Humans show all kinds of reciprocal actions, even with long intervals between helping another and benefitting oneself; thus, humans are rated with (4) concerning reciprocal actions. Rats show direct reciprocity based on their own experience with the other individual, across multiple individuals and over longer time periods (Kettler, Schweinfurth, & Taborsky, 2021; Rutte & Taborsky, 2008). However, there is no evidence of indirect reciprocal actions or the ability to consider third‐party information, leaving rats with an evaluation of (2).

The last feature of social coordination is *prosociality* while we understand it as the ability of an individual to accept costs while helping another individual or cooperating with the other agent. Human children, at 19 months of age, show costly helping behaviour towards strangers, as they hand them their piece of food even though they are hungry themselves (Barragan, Brooks, & Meltzoff, 2020). Adults also show similar behaviour during times of scarce resources, e.g. after a war. Some humans even take the risk of being killed e.g. for hiding political refugees and defending innocent people. These extreme behaviours can even be independent of kinship or close relationships. Thus, humans are willing to accept high costs concerning the relation to others and thus are rated with (4), even though the average person doesn't dare to risk his or her life for anonymous others. Rats also seem to take costs for helping others in need. But since the interpretation of cage‐freeing behaviour is highly disputed (see Section V.6*a*), we evaluate rats with (2).

Summing up over all abilities of social coordination, humans reach a 4 while rats attain a 2.4 [(2 + 2 + 4 + 2 + 2)/5 = 2.4].

### (6) Comparing empathy profiles

Using our evaluation tool, we developed empathy profiles for humans, rodents [rats (*Rattus rattus*, *Rattus norvegicus*) and mice (*Mus musculus*)], apes [chimpanzees (*Pan troglodytes*), bonobos (*Pan pansicus*) and orangutans (*Pongo pygmaeus*)], canids [dogs (*Canis familiaris*) and wolves (*Canis lupus*)] and corvids [ravens (*Corvus corax*), New Caledonian crows (*Corvus moneduloides*) and jays (*Aphelocoma californica, Garrulus glandarius*)]. As described, in the case of (adult) humans for all dimensions, the relevant abilities are strongly present and this evidence from a species‐perspective is common sense and rather easily accessible. Thus, we focus on presenting core relevant evidence for all the other selected animal families. We do this by offering for each relevant ability between one and four references which justify our evaluation of the level of presence of an ability for a species (see Table 2). Looking at the profiles we can easily observe similarities and differences that cannot always be accounted for by one profile being a subclass of the other. This proves the fruitfulness of our approach; we can account for the plurality of types of empathy and their complex interrelations which are not organized like a ladder with increasing complexity. This also allows us to integrate debated cases. We elaborate this further by discussing three case studies.

#### (a) Case 1: comparing rats and humans: can the helping behaviour of rats be classified as empathetic behaviour?

While humans appear to be multi‐talented with regard to empathy, scoring highest in each domain, the profile of rats is more diverse and much less developed in many respects. While they are quite oriented towards others (2.4), they do not do exceptionally well in registering their affective states (2.0) and registering complex situations of others (1.2). Furthermore, evidence for cognitive perspective‐taking is particularly scarce (0.4). These results together with their high flexibility score (3.2) indicate that rats have a potential to go beyond mere selfish desires for social interaction when realizing the cage freeing behaviour. What can we learn about empathy in rats from our way of comparing species? If researchers distinguish types of empathy, these distinctions are often dichotomous, e.g. there is the established difference between *affective* and *cognitive* empathy (for an overview see: Shamay‐Tsoory *et al*., 2009; Yu & Chou, 2018). Given the deficit concerning cognitive perspective taking in rats compared to humans (at least given the studies for now), one may be tempted to claim that rats employ some kind of more affective empathy while humans are able to realize full‐blown empathy integrating both on complex levels. But this is too coarse‐grained because it ignores that rats still have reasonable levels of cognitive abilities as measured by flexibility of their behaviour as well as interesting levels of social coordination of their behaviour. Another option would be to classify rats with these data within the Russian doll model of de Waal & Preston (2017). But, in that model, perspective taking and targeted helping are within the same box. However, we observe that whilst rats have only very rudimentary scraps of (cognitive) perspective taking, they have highly developed targeted helping, suggesting that these abilities do not necessarily go together. Thus, this sequential model is also too coarse‐grained.

We propose that our profile with five dimensions has an adequate detailedness for an informative comparison of empathy in humans and rats. We thereby can overcome the extreme interpretations of the cage freeing behaviour, namely to either adopt a romantic view that rats are empathetic in the same way as human adults are (Bartal *et al*., 2011) or a killjoy view that the observed behaviour was only caused by a ‘selfish motivation’, that is to search for social interaction with the cage mate (Heslin & Brown, 2021). The latter is inadequate since other studies found opposing data for this more parsimonious interpretation (Cox & Reichel, 2020). How can our multidimensional profile approach shape and inform future research? Currently, traditional approaches aim to verify one of these two radically opposing explanations by ruling out the alternative; both are presupposing one main motivation for the behaviour while there is evidence for multiple motivators (Silva *et al*., 2020). Our approach, however, offers a new way of interpreting the observations, *by embedding the rat's helping behaviour into the cluster of relevant abilities* which are represented in the profile of empathy. Consequently, we can make easily visible substantial differences of the same local type of behaviour and avoid systematic overly rich interpretations of rat behaviour. Although the cognitive abilities underlying cage‐release‐activity of rats are still disputed, the evidence indicates that it more than just a product of the selfish motivation for social interaction. But it also remains different from sophisticated forms of empathetic helping in adult humans because the rats' behaviour mainly relies on registering affective states and simple situations rather than on cognitive perspective taking, while humans can rely on all three registration perspectives. This allows humans to adapt to complex social situations involving all kinds of perspective‐taking. Given his difference, we predict less appropriate response behaviour of rats in more complex situations demanding a helping response. Our multidimensional approach thus enables us to pinpoint cognitive differences between species not detectable when just focusing on one behavioural ability. Indeed, in our framework, it is possible that full‐blown empathetic behaviours demonstrated in humans arise from psychological processes similar to those in rats, but that in some situations the human case may include additional components that are absent in the rat case. Thus, rather than a false dichotomy, we expect partial overlap in the component processes that underlie empathy in humans and rats. But it also involves the consequence that the same helping behaviour receives a different evaluation in rats and in humans because it is embedded in quite different cognitive profiles.

Let us now take a short look at the three questions we started out with. Firstly, what is empathy? We suggest a Wittgensteinian characterization of empathy on the basis of the proposed minimal condition: an agent (*A*) is registering another agent's (*B*) affective state in a certain way *x* and the behavioural reaction *r* of *A* is directly related to this registration and is realized in a certain way *y*. Furthermore, we can now specify that, in addition to the minimal condition, we observe a case of empathy if the type of registration *x* satisfies at least an intermediate level (‘weakly present’) of registering the others' affective state or registering the situation or registering the others' mental states (mindset) and if the related reaction of type *y* satisfies at least an intermediate level of flexibility and social coordination. The next question is the *distribution question*: are rats able to act empathetically? Given the profile of rats, the minimal demand is satisfied (they can register the other's affective state to a visible degree and score more than medium level at least on flexibility and social coordination of their behaviour); thus, we are justified to say that rats are able to employ empathetic behaviour. Thirdly, what kind of empathy do rats have? The *quality question* can be answered by pointing to the details of the profile. Since this relates to a sequence of testable abilities it comes with descriptions and predictions of the behavioural and cognitive profile of the empathetic behaviour of a species member. The helping behaviour of rats is evaluated as realized in the context of the rat's profile and thus is evaluated differently than the same helping behaviour in dogs or great apes which have a much more complex cognitive profile. But it also makes visible some overlapping dimensions.

This comparison is developed for humans, rodents (rats and mice), apes (chimpanzees, bonobos and orangutans), canids (dogs and wolves) and corvids (ravens, New Caledonian crows, and jays). Looking at the profiles of corvids and canids we can see in Fig. 3 that the profiles can vary such that we do not always find simple subclasses of each other. This confirms the fruitfulness of this approach; we can account for the plurality of types of empathy which are not organized like a ladder with just increasing complexity. This makes our account much more species‐sensitive than all existing accounts. Based on a profile, we can classify and predict and thereby (to some degree) explain the behaviour of a species regarding empathy.

**Fig. 3:**
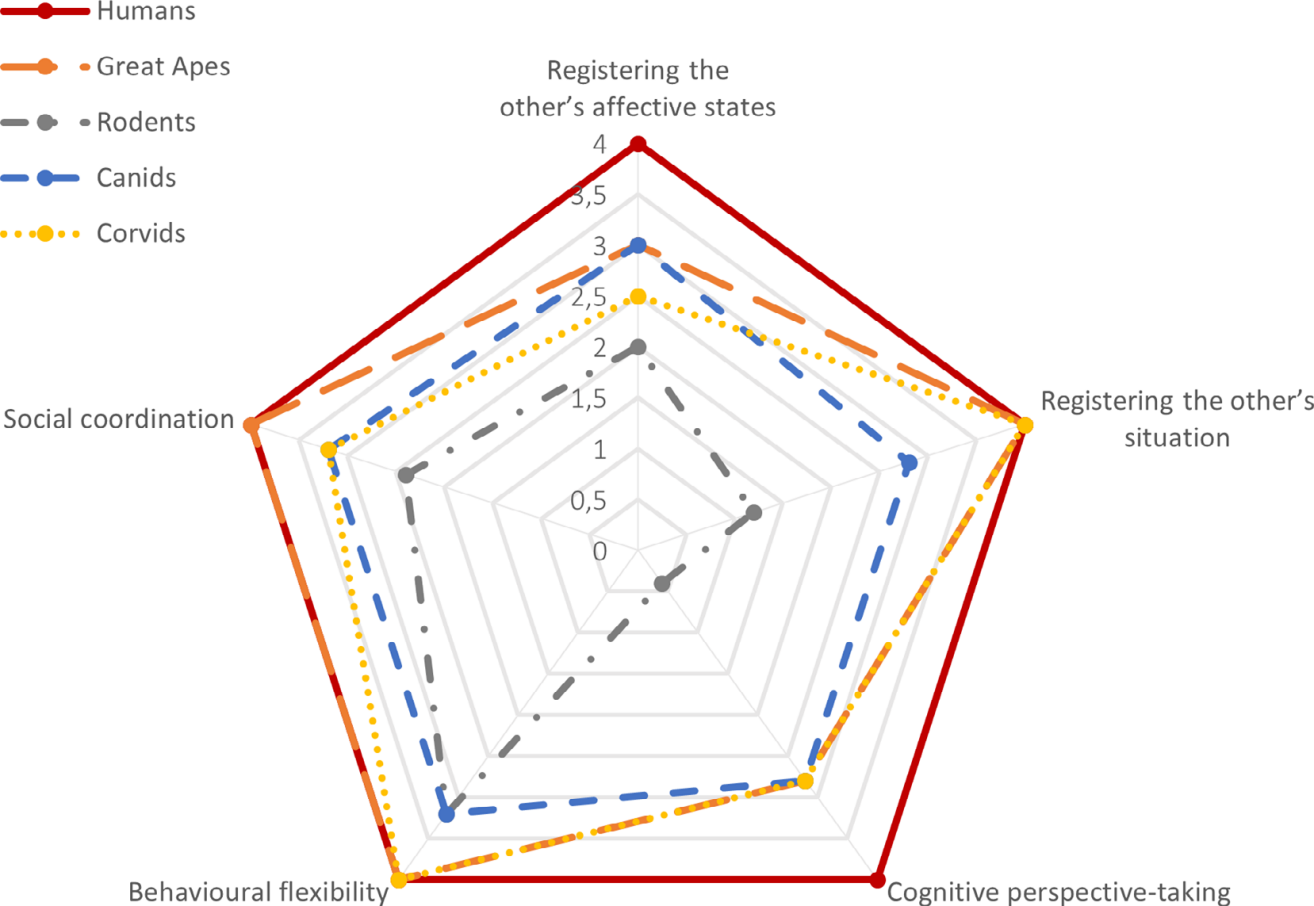
Comparison of the empathy profiles for five species concerning the degree to which they realized the relevant dimensions of empathy.

#### (b) Case 2: comparing canids and corvids: evolutionarily rather distant species with rather similar profiles of empathy

During evolution there was an early bifurcation of a lineage leading to birds and a different one leading to mammals. Both have strongly different ecological challenges due to relying on flying in contrast to running on earth (with a few exceptions) and different brain structures since only mammals developed a laminated cortex. Thus, we would also expect rather different cognitive abilities. And, of course, there are differences, especially related to the abilities of flying, e.g. outstanding visual abilities of crows, and well‐known outstanding smelling abilities in the case of canids. Thus, one might expect large differences concerning empathetic abilities too. But, due to convergent evolution, especially since both families include social species, our profile comparison enables us to see that the relevant cognitive abilities of these social species are rather similar, and thus we also predict empathetic behaviour to be similar too.

Unexpected similarities in canids and corvids can be observed when developing a detailed comparison, as outlined here: emotional contagion can be observed in canids (Huber *et al*., 2017; Palagi, Nicotra, & Cordoni, 2015; Sümegi, Oláh, & Topál, 2014; Van Bourg, Patterson, & Wynne, 2020; Yong & Ruffman, 2014) and corvids (Osvath & Sima, 2014); both show consolation behaviour (ravens: Fraser & Bugnyar, 2010; dogs: Cools, van Hout, & Nelissen, 2008; Kujala, 2017; Quervel‐Chaumette *et al*., 2016). Furthermore, dogs can suppress their own distress and can quickly help caged individuals if they are distressed rather than neutral (Sanford, Burt, & Meyers‐Manor, 2018). Azure‐winged magpies (*Cyanopica cyana*) show proactive prosociality (Horn *et al*., 2016) and especially females share even their favourite food if the other bird lacks food (Massen, Haley, & Bugnyar, 2020).

#### (c) Case 3: comparing humans with other great apes: fine‐grained differences in an enriched dimensional approach

Using our empathy profile, humans and other great apes appear to be very similar in their empathetic profiles. This assessment is in line with many types of empathetic behaviour observed in humans and great apes, such as consolation behaviour (in humans: Hoffman, 2000; in great apes: Romero, Castellanos, & de Waal, 2010) and active helping behaviour (in humans: Buttelmann *et al*., 2009; in great apes: Buttelmann *et al*., 2017). However, research also shows that there seem to be clear species differences concerning complex forms of empathy such as future‐directed empathy. Imagine a human adult observing a child happily playing in the sandbox despite having lost an arm in an accident. The human can abstract the happy situation in the here‐and‐now from problems arising in the future due to the handicap and might feel sorry for the child. Currently, we do not have any evidence of such (long‐term) future‐directed empathy in apes. Such an observation can now be accounted for with our model by zooming into the relevant dimension of cognitive perspective taking. This fine‐grained look enables us to pinpoint specific relevant features and shows that *future‐directed thinking beyond the current situation* and *second‐order theory of mind* (Perner & Wimmer, 1985) do not seem to exist in other great apes. Consequently, future‐directed empathy is not predicted for other great ape species. To test this prediction, we need to add these features into the dimension of cognitive perspective taking and give them a special weight compared to the other features of this dimension. This would result in being able to distinguish fine‐grained differences between empathetic skills in humans and other great apes.

## VI. SITUATING THE MODEL: CHARACTERISTICS AND ADVANTAGES

With the multidimensional profile of empathy, we can develop testable behavioural features that result in a dimensional characterisation of empathetic behaviour. Our aim is to develop these dimensions into a species‐sensitive account. Such an account must start by examining standard human cases of empathy and how they manifest. However, to avoid a strong anthropocentric perspective, we first characterise typical cases by identifying central dimensions of empathy and modify them to account for testing nonhuman animals. To evaluate the behavioural response, we exchanged the typically human dimension of ‘attitude of caring for others’ with the combined dimensions of ‘flexibility’ and ‘social coordination’ (other‐directedness of the response behaviour) in non‐human animals. This transforms the anthropocentric perspective into a more behaviour‐based, species‐sensitive one. An analogous approach has also been applied to consciousness by Birch *et al*. (2020) and Dung & Newen (2023). Rather than proposing a static account of dimensions and features, we are open to developing them further by integrating species‐sensitive aspects based on new evidence. More precisely, we now start with a human case and adopt the relevant dimensions for non‐human animals. Given our starting point, it may be possible in the long run to establish rats or crows as models of intermediate‐level empathy. If we then observe a more species‐specific expression of empathy in rats or birds, this could justify the addition of new testable features or even completely new dimensions, enabling us to continue searching for empathetic behaviour in more distant animals, such as fish or insects. Ideally, the multidimensional profile account is the result of systematically switching back and forth between different perspectives accounting for new evidence to establish the most fruitful behaviour‐based dimensions with which to characterise varieties of empathy (Dung & Newen, 2025).

Furthermore, detailed observations of dimensions and features can broaden our understanding of empathetic behaviour in different species, as well as supporting the search for neural underpinnings. If we were to discover such underpinnings, we would be faced with the question of whether they all share a common computational mechanism, with only different implementations. We will leave this question unanswered, but we believe that cognitive performance always depends on two factors: internal mechanisms and environmental conditions (the ecological niche) that enable and support the performance realised by an agent (Newen, de Bruin & Gallagher, 2018). Thus, we propose that our behavioural‐cognitive approach, which unfolds in a multidimensional profile, is complementary to the search for mechanisms in neuroscience and can also inspire new approaches to the latter.

Another advantage of our approach is that it can be adapted to enable fine‐grained comparisons within species by integrating some or all features of a dimension into the profile comparison. For example, if we want to describe ontogenetic differences in humans fruitfully (e.g. early infancy, schoolchildren and adults), we should treat the features of the cognitive perspective‐taking dimension (i.e. VPT 1 and 2, registering the desires of others and theory of mind ability) as independent dimensions and add different ways of testing these. This would highlight the ontogenetic differences much more effectively. Thus, our dimensions are pragmatically chosen to provide the most fruitful comparative perspective. Finally, although our multidimensional account highlights gradual differences and familiarity between profiles while excluding strict borders, there are still reasonable lower bounds. It does not make sense to speak of empathy in cases where a species is only able to perform motor mimicry in relation to the affective state of another (e.g. mirroring an other's yawn). If there is no evidence of more specific sensitivities or reactive behaviour directed not only at expressive motor behaviour, but also at the associated affective state, then this is an interesting motor ability, but not a case of empathy. This is captured in our minimal condition, which we started with, since it includes registering the other's affective state. We can highlight some prototypical cases of empathy as central to the landscape of empathetic behaviour (Newen *et al*., 2022), provided this is not interpreted by the ‘ladder view’ of empathy as a linear systematic progression. When applying this model, we can describe the similarities and differences between these varieties of empathetic behaviour and related phenomena. For example, according to our view, prosocial behaviour is not inherently empathetic, but rather a feature of the dimension of social coordination, thereby indicating empathy. Finally, the account enables a systematic investigation of evolutionary comparisons between two species, as well as of ontogenetic development within one species. It especially enables us to distinguish between similar consolation behaviours, considering their placement within relevant cognitive dimensions. For example, there is consolation behaviour within an ape group based on recognition of anger after losing a fight, and a complex human case of consolation involving anticipation of future sorrow with advanced perspective‐taking for an agent who is not currently suffering.

## VII. CONCLUSIONS

(1) We offer a *new multidimensional profile account* to describe empathy in human and nonhuman animals from a comparative perspective. It involves five dimensions and several testable features indicating, respectively, whether and to which degree one dimension is realized.
(2) It answers three important questions, namely how to fruitfully characterize the phenomenon, whether a certain species has empathy at all (the distribution question) and which variant of empathy it is able to realize (the quality question).
(3) It is a new strategy for overcoming an impasse of empirical results concerning cognitive ability, illustrated with the case of the empathetic helping behaviour in rats.
(4) It proposes a less anthropocentric perspective on the behaviour of nonhuman animals by modifying the relevant dimensions (which start from a human example) to be less intellectual and more open to species‐specific biology and behaviour.
(5) By offering a framework that enables us to systematically compare different species without simplified ‘yes/no’ answers to the question of empathetic behaviour, we can discover unexpected differences and similarities: empathetic behaviour in canines and corvids is apparently not too different.
(6) A behaviour of an individual agent is always evaluated in the context of agent's cognitive‐behavioural profile that is relevant for empathy. The same behaviour realized in different species can receive a different evaluation concerning its degree of being empathetic if they are embedded into different cognitive profiles.
(7) The multidimensional profile account could allow comparison of mammalian species with non‐mammalian species like birds, fish and invertebrates since the comparison need not rely on similarity in neural organization but rather focuses on the comparison of the behavioural‐cognitive profile.
(8) The multidimensional profile (MP) account is complementary to all the approaches which directly aim to discover underlying mechanisms of empathy. Neuroscientific investigations, psychophysical studies, etc. which aim to identify the underlying mechanisms are complementary in both directions; on the one hand, they help to find good candidates for dimensions of a cognitive ability for an MP account, on the other hand, the MP account aims to find predictions for new investigations of the underlying mechanisms. Thus, the joint final aim is the discovery of underlying mechanisms in a species‐specific way.
